# Comparison of an oblique single cut rotation osteotomy with a novel 3D computer-assisted oblique double cut alignment approach

**DOI:** 10.1038/s41598-021-94141-4

**Published:** 2021-07-19

**Authors:** Johannes G. G. Dobbe, Peter Kloen, Simon D. Strackee, Geert J. Streekstra

**Affiliations:** 1 Department of Biomedical Engineering and Physics, Amsterdam Movement Sciences, Amsterdam UMC, University of Amsterdam, Room no L0-113-3, Meibergdreef 9, 1105 AZ Amsterdam, The Netherlands; 2 Department of Orthopedic Trauma Surgery, Amsterdam Movement Sciences, Amsterdam UMC, University of Amsterdam, Meibergdreef 9, Amsterdam, The Netherlands; 3 Department of Plastic, Reconstructive and Hand Surgery, Amsterdam Movement Sciences, Amsterdam UMC, University of Amsterdam, Meibergdreef 9, Amsterdam, The Netherlands

**Keywords:** Health care, Engineering, Mathematics and computing

## Abstract

An oblique double-cut rotation osteotomy (ODCRO) enables correcting a complex bone deformation by aligning, in 3D, the distal, middle and proximal bone segments with a target bone, without intersegmental gaps. We propose virtual preoperative planning of an ODCRO. To minimize a residual translation error, we use an optimization algorithm and optimize towards bone length, alignment in the transverse direction, or a balanced reconstruction. We compare the residual alignment error with an oblique single-cut rotation osteotomy using 15 complex bone deformations. The single-cut approach was not feasible in 5 cases, whereas the ODCRO procedure was feasible in all cases. The residual alignment error was smaller for the ODCRO than for the single-cut approach except for one case. In a subset for length reconstruction, the length error of 7.3–21.3 mm was restored to 0.0 mm in 4 of 5 cases, although at the cost of an increased transverse translation. The proposed method renders planning an ODCRO feasible and helps restoring bone alignment and lengthening better than an oblique single-cut rotation osteotomy. Awareness of the challenges and possibilities in preoperative planning of an ODCRO will be of value for future alignment surgery and for patients.

## Introduction

Fracture management may lead to symptomatic malunion of bone segments, requiring surgical treatment. An established treatment option is a corrective osteotomy^1^. Several osteotomy types exist such as the opening wedge osteotomy, in which a bone is cut and a segment is tilted to improve alignment, leaving an open wedge, which is either filled^2^ or not filled with a bone graft^3^. The alternative is using a closing wedge osteotomy where a wedge is removed and the wedge-shaped gap is closed. A disadvantage of the closing-wedge osteotomy is the obvious bone shortening.

When rotational deformity (torsion) coexists with angular deformity, correction can be achieved using an oblique *single*-cut rotation osteotomy (OSCRO), in which the obliquity of the single cut is planned in a specific direction^4–6^. Subsequent rotation of the distal bone segment about the axis perpendicular to the oblique osteotomy plane yields rotational alignment in the sagittal, coronal and axial planes without bone loss, while maintaining bone contact^4^. However, when rotational deformity is small, a very steep oblique cut is required^4^, which is usually not clinically feasible. An OSCRO also fails if a bone deformity extends over a particular length, for example due to trauma or disease^7^. In these cases the procedure may result in a local irregularity where the oblique osteotomy is placed. In cases where an OSCRO fails, a better treatment option may be an oblique *double*-cut rotation osteotomy (ODCRO).

In an ODCRO two osteotomies are performed as in a closing wedge osteotomy, but the wedge is reused as an autologous bone graft, in such way that alignment is achieved while the bone segments stay in contact. By choosing osteotomy planes with appropriate obliquity and adequate bone rotations, one can plan perfect rotational alignment. However, translational malalignment of the bone segments may remain. By choosing an optimal set of correction parameters (osteotomy plane locations, osteotomy plane orientations, bone-segment rotations, in-plane bone-to-bone translations) one can minimize residual translations, yielding optimal alignment of the bone segments with a target bone. Preoperatively planning the right set of osteotomy parameters, however, is not a trivial task because several degrees of freedom have to be optimized for spatial adjustment of bone fragments, osteotomy locations and orientations, while taking into account biomechanical and medical constraints^8^.

In this paper we propose an iterative method for optimal planning of an oblique double-cut rotation osteotomy, in which the intermediate bone segment is used as autologous bone graft. We evaluate alignment by simulation and compare it with the achievements of an oblique single cut rotation osteotomy.

## Materials and methods

**Figure 1:**
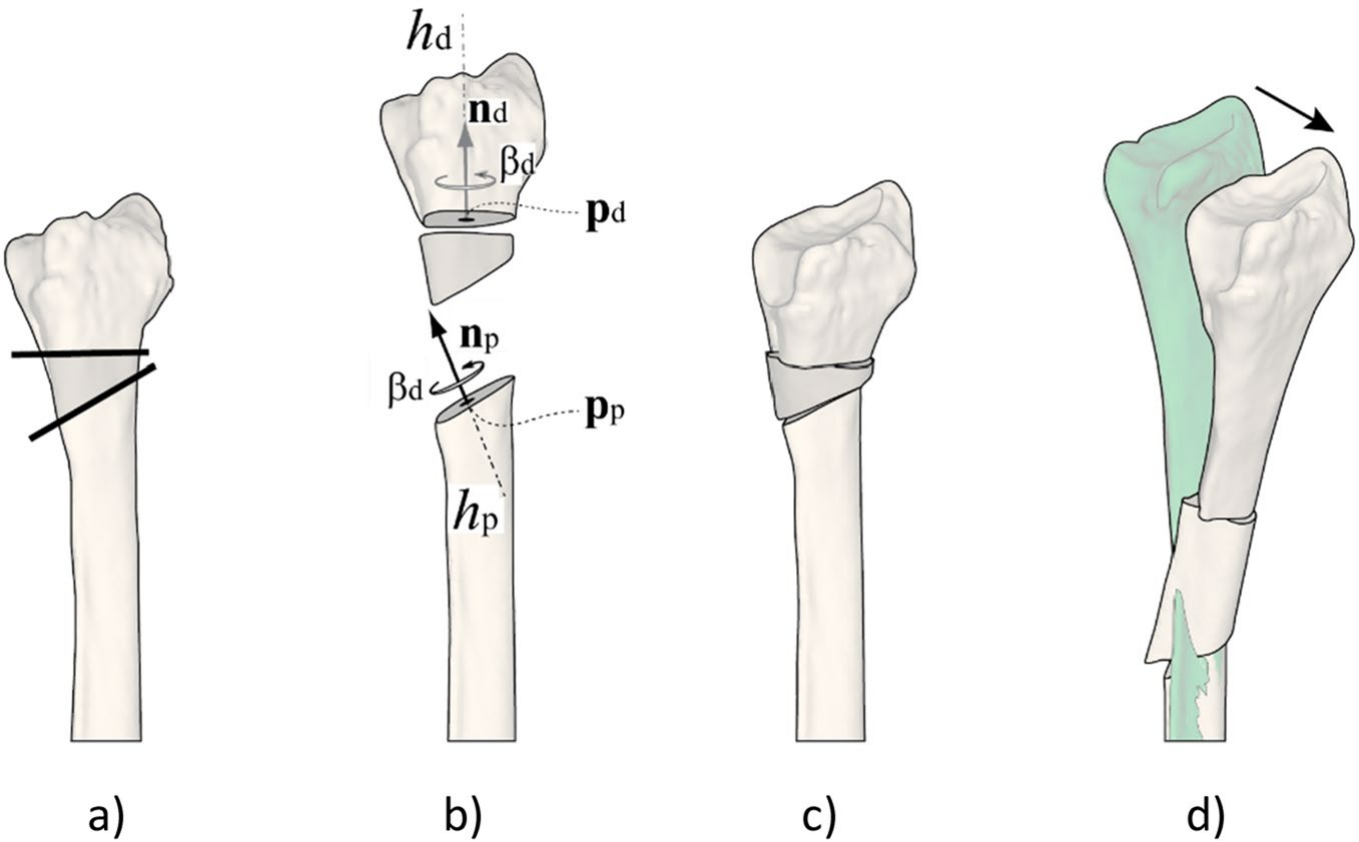
(**a**) Rotational alignment correction of a deformed bone using an oblique double-cut rotation osteotomy. (**b**) The distal segment is rotated over β_d_ about the axis (*h*_d_) oriented in the direction of the cutting plane normal (**n**_d_) and a point on the axis (**p**_d_), being the centroid of the polygon points in the cutting plane. A second axis (*h*_p_) is defined in the same way using the proximal cutting plane (cutting plane normal **n**_p_, point on the axis **p**_p_). The rotated distal segment and the middle segment are considered a single assembly which is rotated about the axis over the angle β_p_. The exploded view shows applicable parameters. (**c**) Aligned bone segments after rotation. (**d**) In general, a residual translation error may persist (arrow) between the corrected bone and the target bone (green), although rotational alignment is achieved. This translation error depends on the chosen osteotomy parameters, such as the osteotomy plane locations.

An ODCRO basically consists of two oblique rotation osteotomies. Figure 1a shows part of a deformed radial bone and defines a distal and proximal cutting plane. Repositioning of the distal bone segment is achieved by first rotating the distal bone over an angle *β*_d_ about axis *h*_d_, which is perpendicular to the distal cutting plane (Fig. 1b). Next, the distal bone segment and the wedge are considered a single unit, which is rotated over an angle *β*_p_ about the second axis *h*_*p*_ perpendicular to the proximal cutting plane to achieve rotational alignment (Fig. 1c). The first challenge in performing an ODCRO is to choose a set of osteotomy parameters, i.e., cutting planes and bone rotations, that restore rotational alignment of the distal bone segment. We will show that this is possible by calculating parameters for one oblique osteotomy (obliquity, bone rotation) once the parameters for the other osteotomy are chosen. However, the aforementioned approach does not take into account the inherent but undesired translation of the distal and middle bone segments (Fig. 1d). The optimal set of osteotomy parameters therefore not only restores rotational alignment but also minimizes residual translations of the bone segments. This optimal set of osteotomy parameters can be found by a user in a manual approach by interactively choosing a first osteotomy plane (location and obliquity) and bone rotation, while an algorithm calculates the same osteotomy parameters for the other plane. If translational alignment is unsatisfactory, the user can manually adapt the first osteotomy parameters and the location of the other osteotomy and let the algorithm calculate its obliquity and the corresponding bone rotation. An alternative to this trial and error approach is using an automatic optimization algorithm to find the optimal osteotomy parameters for both planes. In this paper we describe the theory behind calculating the osteotomy parameters of one plane when the other osteotomy parameters are chosen by the user. We further propose using an optimization algorithm to improve translational alignment of all three bone segments with a target bone.

### Planning of an ODCRO

Performing an ODCRO requires position planning of the distal bone segment with respect to the proximal bone segment. The mirrored contralateral bone serves as planning target (reference) in this study. As a pre-processing step we proximally align the affected and target bones to visualize where the affected bone starts deviating, but also to visually and quantitatively evaluate the result of performing an ODCRO. Since a statistically significant relation was found between rotational malalignment and patient satisfaction^9^ after surgery, our aim was to perfectly plan rotational alignment and to minimize residual translational malalignment. Below we describe the theory behind preoperative position planning, restoring rotational alignment in the application of an ODCRO, and optimizing translational alignment in this approach.

#### Preoperative position planning

**Figure 2:**
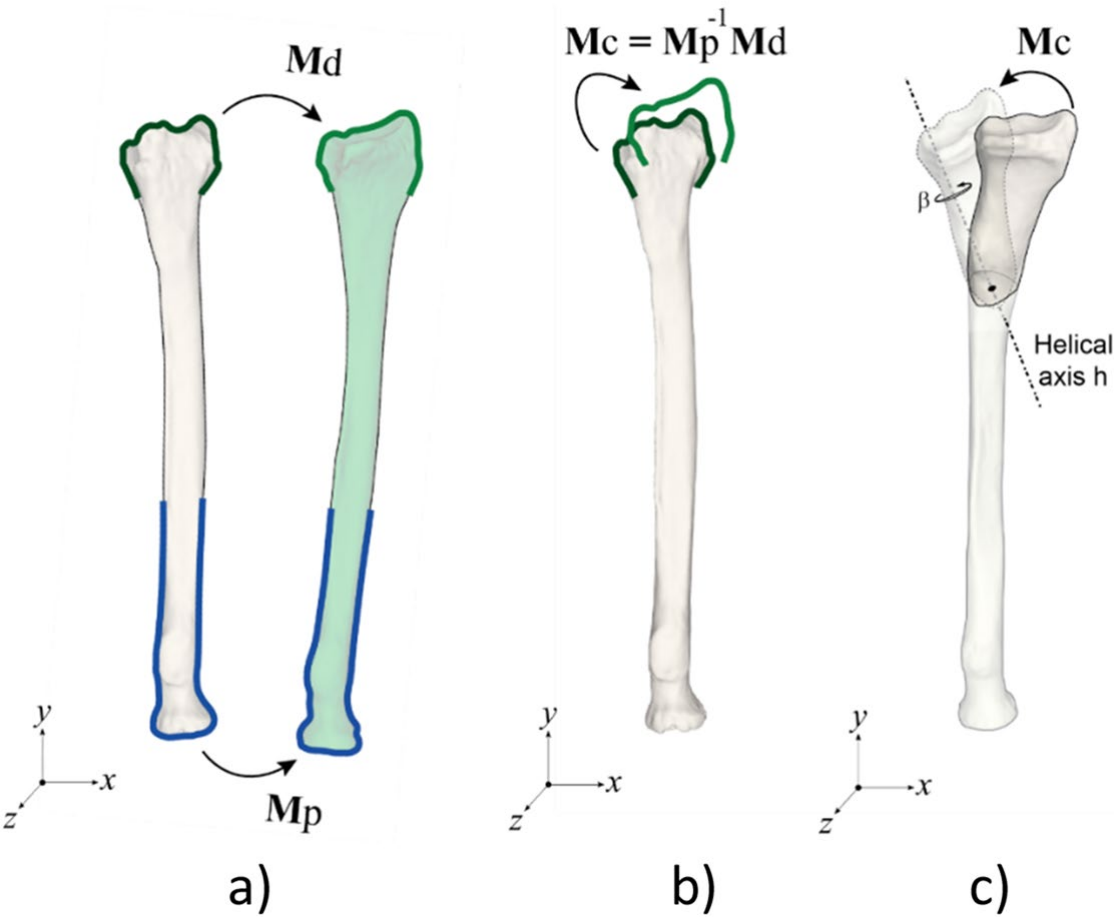
(**a**) Registration of a distal and proximal segment to a target bone yield **M**d and **M**p from which the correction matrix (**b**) can be calculated. (**c**) An oblique single-cut rotation osteotomy (OSCRO) achieves alignment by rotation *β* of the distal segment about the helical axis *h*. The cutting plane is chosen perpendicular to this helical axis.

In this paper we adopt a method for position planning as described before^10,11^. In short, the affected bone is segmented and distal and proximal segments are clipped for subsequent registration to the mirror image of the contralateral healthy bone. The segments were clipped in order to exclude the deformity since this would deteriorate registration to the target image. Registration yields two 4 × 4 matrices for transforming the distal (**M**d) and proximal segment (**M**p) to the contralateral bone (Fig. 1a). The correction matrix (**M**c), which transforms the distal segment from the affected to the planned position in the coordinate system of the preoperative image (Fig. 2) containing the affected bone, can now be calculated:

1 $${\mathbf{M}}{\text{c}} = {\mathbf{M}}{\text{p}}^{{ - {1}}} {\mathbf{M}}{\text{d}}$$

#### Restoring rotational alignment ‘manually’

The standard way to describe bone repositioning in 3-D space is by placing it at the center of an orthogonal coordinate system, rotating it about its axes, and translating it to the desired location. An alternative way to describe this object transformation is by using a helical axis (Fig. 2c)^5^. This axis is positioned in 3-D space such, that the transformation is achieved by rotating the object over an angle, *β*, about the helical axis and translating the object along the helical axis over a distance *d*. The rotational part of this method is used in an oblique single-cut rotation osteotomy (OSCRO)^4–6^, where the cutting plane is chosen perpendicular to the helical axis, and rotating the distal bone segment over the angle *β* yields rotational alignment. The parameter *d* is set to 0, which keeps the bone faces connected but introduces a translation error. An ODCRO basically consists of two OSCRO’s in which specific rotation axes and rotation angles are chosen.

**Figure 3:**
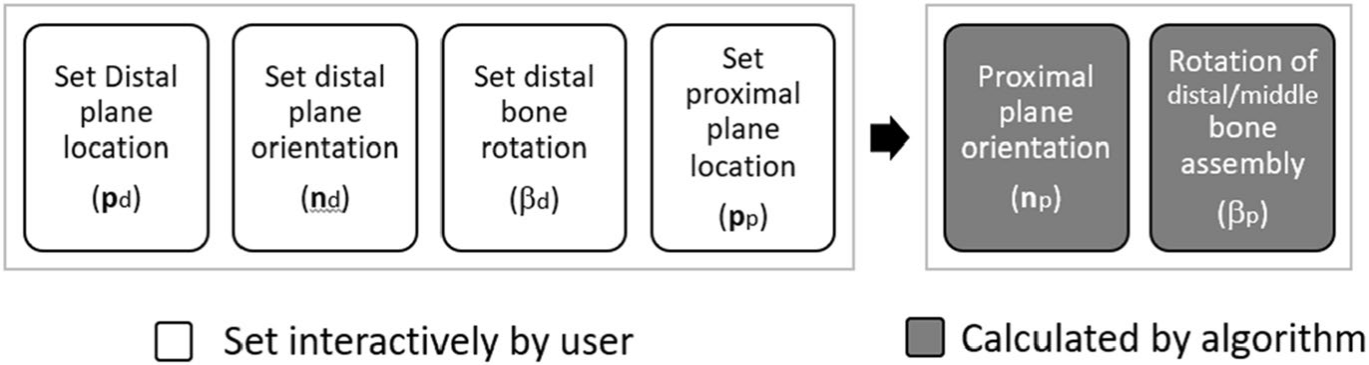
Osteotomy parameters. The distal plane parameters (**p**_d_, **n**_d_, *β*_d_) and the location of the proximal plane (**p**_p_) are interactively set by the user. The orientation of the proximal plane (**n**_p_) and the rotation angle of the distal-middle bone assembly (*β*_p_) are calculated by the algorithm.

**Figure 4:**
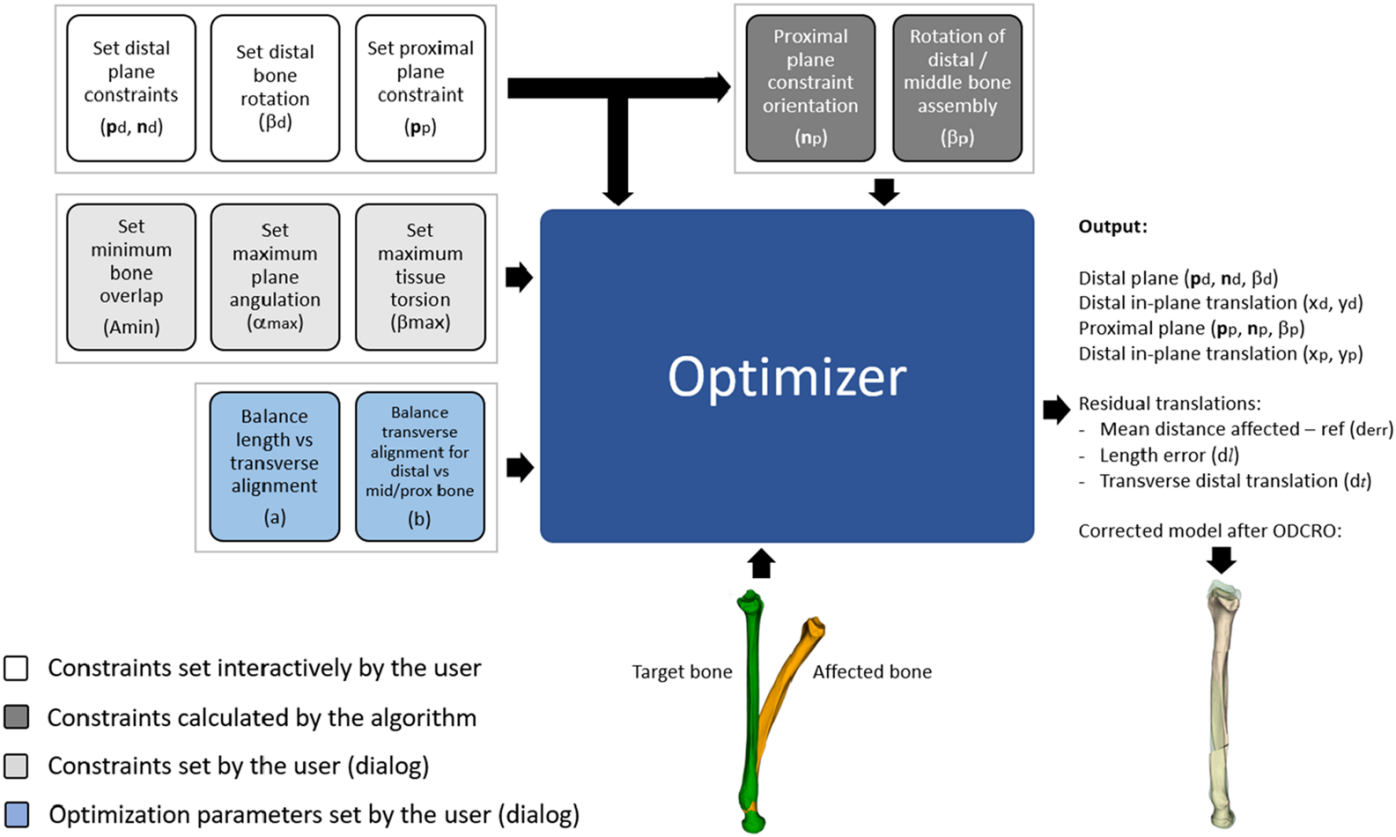
Diagram showing inputs to the optimizer including bone models and parameters set by the user either interactively or through a dialog window. Some input parameters are calculated automatically by the algorithm. The optimizer provides the osteotomy parameters (distal and proximal plane position and orientation; in-plane translations and bone rotations) residual translations, and the corrected bone model.

For planning of an ODCRO, we first focus on restoring rotational alignment using two oblique rotation osteotomies and ignore possible bone translations that we may introduce with the procedure. Repositioning of the distal bone segment in Fig. 1a is achieved by rotating the distal bone over an angle *β*_1_ about axis *h*_d_ (*h*_d_:**x** = **p**_d_ + λ_1_ **n**_d_) defined by the distal cutting plane normal (**n**_d_) and a point (**p**_d_) on that axis. Since we wish to keep the bone faces connected in a double-cut osteotomy, we choose to position the axis through the centroid of the distal cross section (**p**_d_). Next, the distal-middle bone assembly is rotated over an angle *β*_p_ about the second axis *h*_*p*_ (*h*_p_:**x** = **p**_p_ + λ_2_ **n**_p_) defined by the proximal cutting plane normal (**n**_p_) and a point (**p**_p_) on that axis. The centroid of the proximal cross section (**p**_p_) is again used to define the position of the axis. In this procedure we basically performed two axis rotations *β*_d_ and *β*_p_, which jointly restore rotational alignment of the distal bone segment. If we define the distal axis rotation by **R**_d_(**p**_d_, **n**_d_, *β*_d_) (see appendix) and the proximal axis rotation by **R**_p_(**p**_p_, **n**_p_, *β*_p_), the following expression needs to be solved to achieve rotational alignment:

2 $${\mathbf{R}}_{{\text{c}}} = {\mathbf{R}}_{{\text{p}}} \left( {{\mathbf{p}}_{{\text{p}}} ,{\mathbf{n}}_{{\text{p}}} ,\beta_{{\text{p}}} } \right){\mathbf{R}}_{{\text{d}}} \left( {{\mathbf{p}}_{{\text{d}}} ,{\mathbf{n}}_{{\text{d}}} ,\beta_{{\text{d}}} } \right),$$

where **R**c is the top-left 3 × 3 rotation matrix of matrix **M**c (Eq. 1)^12^. There are multiple solutions to solve this equation. Choosing the distal cutting plane, (**p**_d_, **n**_d_) interactively, and choosing the angle *β*_d_ to rotate the distal bone segment, defines **R**_d_ (see appendix). Then, **R**_p_ can be calculated using **R**_p_ = **R**_c_**R**_d_^-1^. The orientation of the proximal cutting plane **n**_p_ and the rotation angle *β*_p_ are finally calculated by^5^:

3 $${\mathbf{n}}_{p}=\frac{1}{2Sin\left({\beta }_{p}\right)}\left|\begin{array}{c}{r}_{23}-{r}_{32}\\ {r}_{31}-{r}_{13}\\ {r}_{12}-{r}_{21}\end{array}\right|$$

4 $${\beta }_{p}=arccos\frac{{r}_{11}+{r}_{22}+{r}_{33}-1}{2}$$

with *r*_*ij*_ the matrix element of **R**_p_ at row *i* and column *j*. In the above description we chose parameters for the distal plane (**p**_d_, **n**_d_, *β*_d_) to define **R**_d_ and calculate **R**_p_. Note that the same reasoning is valid for calculating **R**_d_ and the parameters of the distal plane if we wish to choose the parameters of the proximal plane (**p**_p_, **n**_p_, *β*_p_) to define **R**_p_. For reasons of simplicity we assume in this paper that the distal plane parameters are set by the user interactively (or automatically by the optimization algorithm) and the proximal plane parameters are calculated by the algorithm as visualized in Figs. 3 and 4. In practice the user may choose to position one plane to not interfere with soft tissue structures and subsequently check if the other plane is feasible as well at the same time judging whether the residual translational error (see Fig. 1d) is acceptable. This trial-and-error method is cumbersome, hence the approach with automatic translation optimization.

#### ODCRO with automatic translation optimization

**Figure 5:**
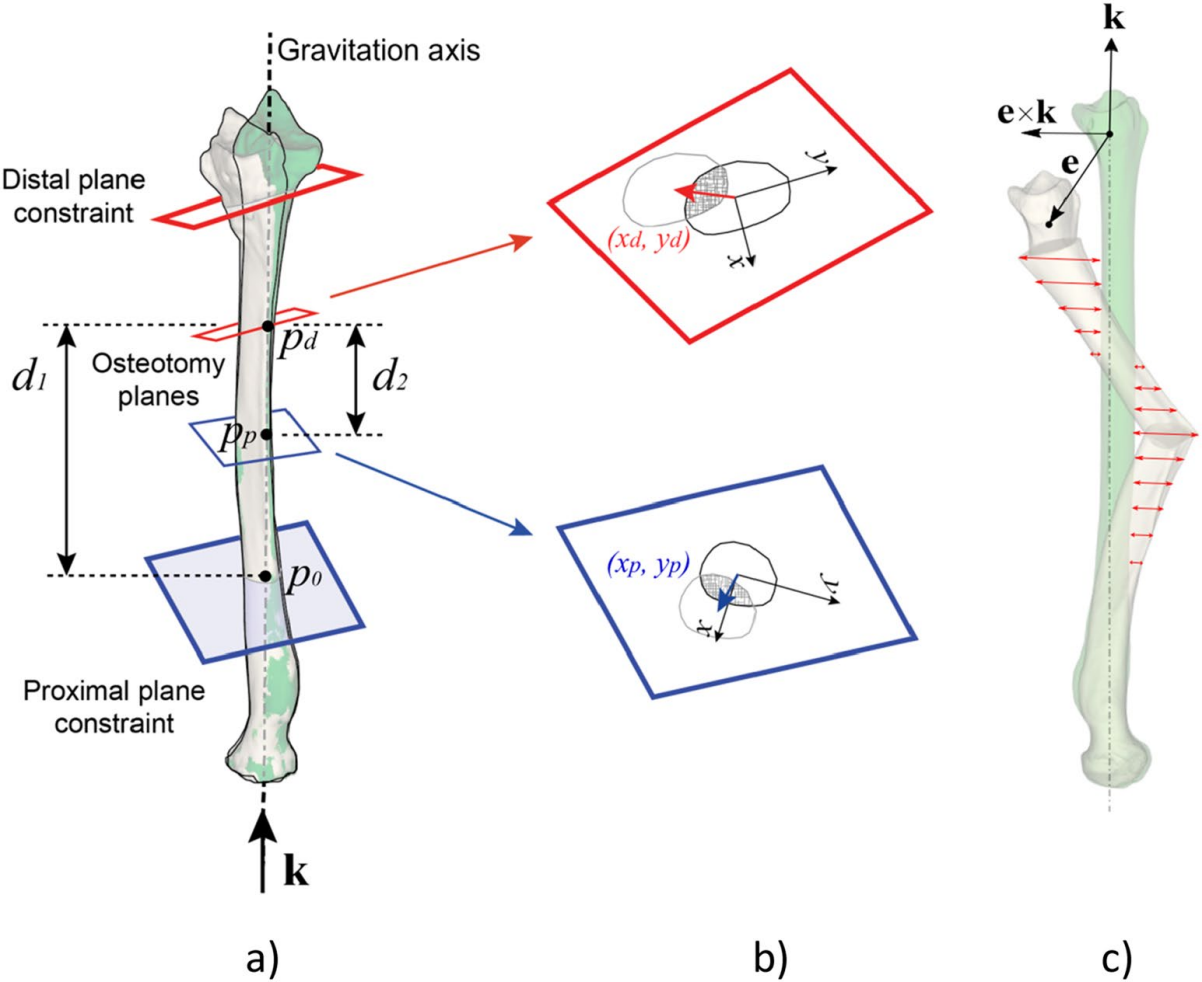
Definition of the parameters involved in optimizing translational alignment of an affected bone with a proximally aligned target bone (green). (**a**) The direction of the gravitation axis of the target bone is represented by unit vector **k**. During the optimization procedure the distal and proximal plane positions are defined by (*d*_*1*_*, d*_*2*_), as measured along the gravitation axis of the bone. The large planes define the search range for the osteotomy planes and are set by the user. The orientation of a distal search plane (**n**_d_) is quantified by azimuth and elevation angles (*φ*_*d*_, ψ_d_) and the rotation of the distal segment is equal to *β*_d_ (see Fig. 1). The proximal plane orientation and bone rotation follow by calculation (**R**_p_ = **R**_c_**R**_d_^−1^ and Eqs. 3 and 4). (**b**) In-plane translations of the distal and proximal bone segments are defined in the sideward and upward (*x, y*) direction of the bone cross section. The shaded surface areas represent the amount of bone overlap. (**c**) Vector **e** represents the residual translation error of the distal bone segment and is used to calculate the error in the length and transverse direction (see text). The mean of the nearest-neighbor distances (red lines) between mesh points of the middle-proximal bone assembly and the target bone (green) is used to control alignment of these bone segments (*e*_*m*_ in Eq. 5).

**Figure 6:**
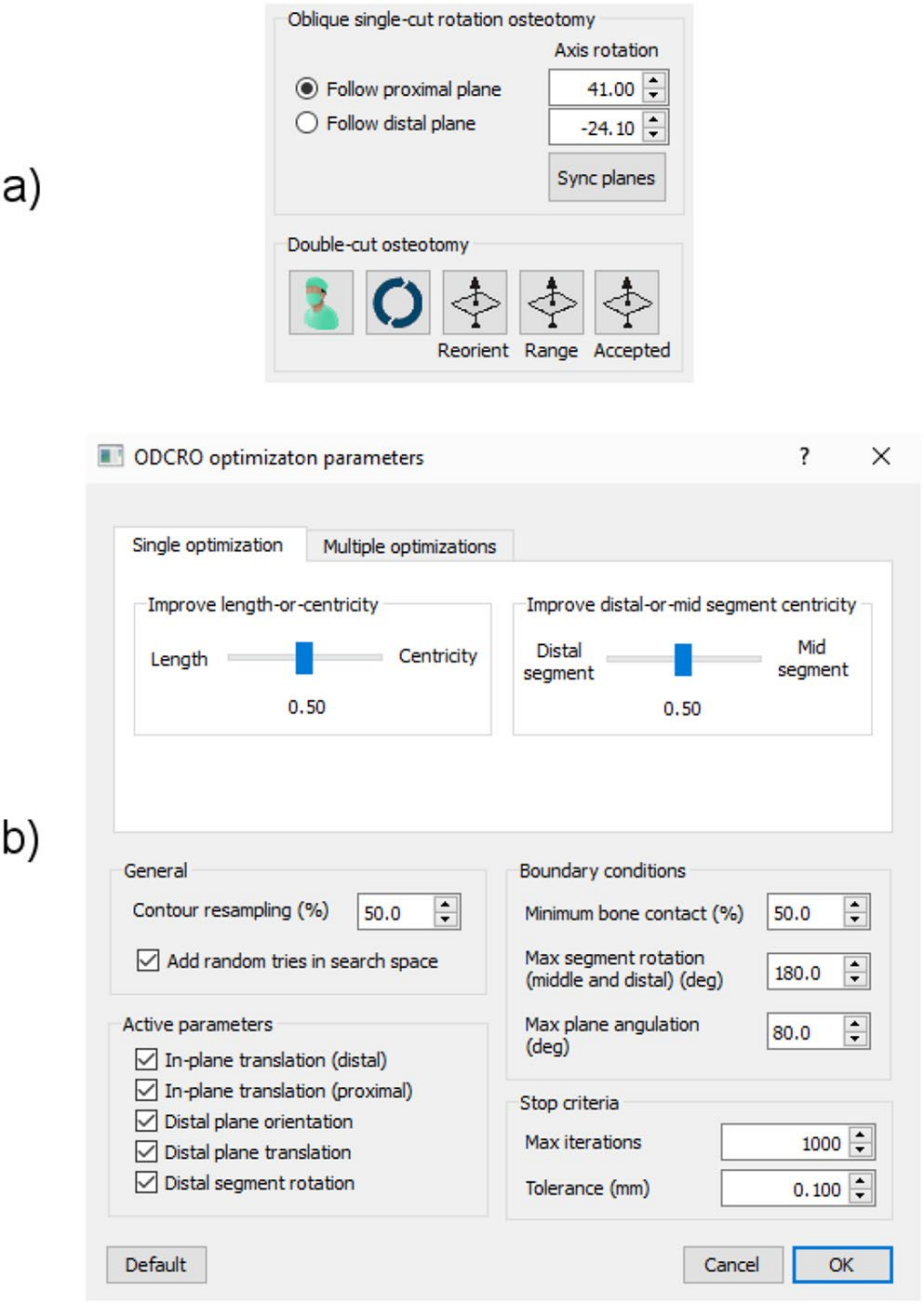
(**a**) User interface to control performing an oblique double-cut rotation osteotomy manually, or, (**b**) to set the optimization parameters for the automatic procedure.

The approach in the previous section restores rotational alignment but does not take into account the translation that the bone segments undergo when they are rotated about the rotation axes (Fig. 1d). Choosing different plane locations, orientations and bone rotations may result in smaller translation errors. To find an optimal set of parameters (**p**_d_, **n**_d_, *β*_d_, **p**_p_, **n**_p_, *β*_p_) (Fig. 1b) we have implemented a modified downhill simplex optimizer by Nelder and Mead^13^ and iteratively compare bone segment repositioning after virtual application of an ODCRO with the target bone. To reduce the chance of ending up in a suboptimal minimum, the modified algorithm alternates between standard simplex optimization^13^ and random tries bounded by the search space. In our optimization approach we adopt in-plane translation of the bone segments as well (*x*_*d*_*, y*_*d*_) and (*x*_*p*_*, y*_*p*_), in an attempt to further improve positioning. The user initializes the procedure by setting distal and proximal plane constraints (Figs. 4, 5). The optimization algorithm is allowed to search for an optimal set of osteotomy parameters within this region. Other constraints and optimization choices, which will be discussed below and are listed in Table 1 and Fig. 4, are set by the user through a dialog window (Fig. 6) just before the optimization procedure starts.

**Table 1:** (a) The osteotomy parameters which are involved in the application of an ODCRO and their relation with (b) optimization parameters visualized in Figs. 1b and 5. In this example the distal plane is chosen by the algorithm, the proximal plane parameters can then be calculated. (c) Shows how these optimization parameters are calculated.

Description	(a) Osteotomy parameter	(b) Optimization parameter	(c) Calculation of optimization parameter based on osteotomy parameter
Distal plane location	**p**_d_	d_1_ (*)	d_1_ =|**p**_d_—**p**_0_|
Distal plane orientation	**n**_d_	*φ*_d_, *ψ*_d_	Azimuth (*φ*_d_) and elevation (*ψ*_d_) angles: $${\varphi }_{d}=\mathrm{arctan}\left(\frac{{{\varvec{n}}}_{d}(y)}{{{\varvec{n}}}_{d}(x)}\right); {}_{2}=\mathrm{arctan}\frac{{{\varvec{n}}}_{d}(z)}{\sqrt{{{{\varvec{n}}}_{d}(x)}^{2}+{{{\varvec{n}}}_{d}(y)}^{2}}}$$
Distal bone segment rotation	*β*_d_	*β*_d_	
Proximal plane location	**p**_p_	d_2_ (*)	d_2_ =|**p**_d_ – **p**_p_|
Proximal plane orientation	**n**_p_	(#)	Azimuth (*φ*_d_) and elevation (*ψ*_d_) angles (**n**_p_, see Eq. 4): $${\varphi }_{p}=\mathrm{arctan}\left(\frac{{{\varvec{n}}}_{p}(y)}{{{\varvec{n}}}_{p}(x)}\right); {}_{2}=\mathrm{arctan}\frac{{{\varvec{n}}}_{p}(z)}{\sqrt{{{{\varvec{n}}}_{p}(x)}^{2}+{{{\varvec{n}}}_{p}(y)}^{2}}}$$
Rotation of distal/middle bone assembly	*β*_*p*_	(#)	See Eq. (3)
In-plane sideward and upward translation of distal bone segment ($)	*x*_*d*_*, y*_*d*_	*x*_*d*_*, y*_*d*_	
In-plane sideward and upward translation of distal/middle bone assembly ($)	*x*_*p*_*, y*_*p*_	*x*_*p*_*, y*_*p*_	

(*) Measured along the gravitation axis of the target bone (see text).

(#) proximal plane follows distal plane (see Eqs. 3 and 4).

($) Measured along the two gravitation axes of the points describing the distal or proximal bone cross section (Fig. 5b).

**Optimization parameters** (Table 1). Given the fact that **p**_d_, **n**_d_, **p**_p_ and **n**_p_ are 3 × 1 column vectors, the total number of optimization parameters (**p**_d_, **n**_d_, *β*_d_, *x*_*d*_*, y*_*d*_, **p**_p_, **n**_p_, *β*_p_, *x*_*p*_*, y*_*p*_) would be 18. However, this can be reduced to a nine-parameter search space of independent parameters using the following reduction strategy. Since **n**_p_ and *β*_p_ can be calculated once a distal plane is chosen (see Eqs. 3 and 4), they do not need to be included as optimization parameters. This saves four optimization parameters. The location of the osteotomy planes (**p**_d_, **p**_p_) can be expressed by distances measured along a line, which saves another four optimization parameters. To this end we calculate and use the gravitation axis of the target bone (Fig. 5a). This axis is determined by calculating the inertia tensor from the polygon mesh points of the target bone. This tensor enables calculating the three eigenvectors and eigenvalues. The unit vector **k** (Fig. 5a) with the smallest eigenvalue identifies the direction of the gravitation axis. The centroid of the target bone (**c**) completes the vector representation of the gravitation axis (*g*:**x** = **c** + λ **k**). The distal and proximal osteotomy planes intersect the gravitation axis at **p**_d_ and **p**_p_. We use the intersection point (**p**_0_) of the gravitation axis and the proximal plane constraint (Fig. 5) as reference in calculating the plane positions, defined by *d*_1_, the distance between **p**_d_ and **p**_0_, and *d*_2_ the distance between **p**_d_ and **p**_p_. Finally, the orientation of the distal osteotomy plane can be expressed in terms of azimuth (φ) and elevation (ψ) angles, which saves another optimization parameter.

**Constraints.** Besides the aforementioned distal and proximal plane constraints, the algorithm takes into account the minimum amount of bone overlap (*δ*_d_, *δ*_p_), the maximum allowed plane angulation (*ρ*_p_, *ρ*_d_) and the maximum amount of tissue torsion (μ_d_, μ_p_). These constraints and the involved parameters are described in Table 2 and affect the metric (detailed below) which gives guidance to the optimizer. The optimizer tries to find a set of osteotomy parameters which leads to a small residual translation of the bone segments, i.e. a low value of the metric. Optimizer attempts which go beyond the set constraints should therefore largely increase the metric, hereby driving the optimization algorithm toward a better solution.

**Table 2:** Penalty calculation.

Constraint	Metric parameter	Penalty equation	Description
Minimum bone overlap	*δ*_*d*_, *δ*_*p*_ (*)	${10}^{{100(A}_{rel}-{A}_{\mathrm{min}})}$	This penalty term increases exponentially once the overlap drops below a user-defined limit (*A*_*min*_).With *A*_*rel*_ the relative amount of bone overlap (range [0,1]) at the distal and proximal interfaces respectively
Maximum plane angulation	*ρ*_p_, *ρ*_d_ (*)	${2}^{\alpha -{\alpha }_{max}}+{2}^{-\alpha -{\alpha }_{max}}$	The steepness *α* is calculated as the angle between the plane normals **n**_d_, **n**_p_ and **k** representing the direction of the gravitation axis. Avoids steep osteotomies by increasing the metric exponentially when the steepness of either osteotomy plane exceeds *α*_max_ as set by the user;
Maximum tissue torsion	*μ*_p_, *μ*_d_ (*)	${2}^{\beta -{\beta }_{max}}+{2}^{-\beta -{\beta }_{max}}$	Increases exponentially when the proposed bone rotation *β* = [*β*_d_, *β*_p_] exceeds a predefined torsion limit *β*_max_ as set by the user
Plane position	*τ*	$100({n}_{a}+{n}_{b}+{n}_{c})$	Increases when either osteotomy plane passes beyond the plane constraints as set by the user. For this evaluation the cross section of the bone is first determined using the proximal and distal plane constraints. This provides two point sets p_1_ and p_2_ *n*_*a*_ is assigned the number of points out of p_1_ that are found distally from the distal plane constraint *n*_*b*_ is assigned the number of points out of p_2_ proximally from the proximal plane constraint *n*_*c*_ contains the number of points in the volumetric overlap that remains after osteotomizing a distal and proximal bone segment using the two osteotomy planes. If the osteotomies do not intersect, *n*_*c*_ equals 0

In cases where the optimizer exceeds a given constraint the penalty largely increases the metric, effectively invalidating the current optimizer attempt.

(*) Metric parameter separately determined for distal and proximal plane, as indicated by the index (*d, p*).

**The metric**. The metric (*ε*) that guides the optimization procedure is defined as follows:

5a $$\varepsilon =\left(1-a\right) {e}_{l}+a{ e}_{t}+\theta$$

5b $${e}_{l}=\left|{\varvec{e}}\cdot {\varvec{k}}\right|$$

5c $${e}_{t}=\left(1-b\right)\left|{\varvec{e}}\times {\varvec{k}}\right|+b{ e}_{m}$$

5d $${\theta =\delta }_{d}+{\delta }_{p}+{\rho }_{d}+{\rho }_{p}+{\mu }_{d}+{\mu }_{p}+\tau$$

Here *a* (range [0, 1]) is set by the user and balances between optimizing the length error *e*_*l*_ and the transverse error *e*_*t*_ of all bone segments. Parameter *θ* adds a high penalty to the metric if any of the constraints (Table 2) is exceeded, which effectively invalidates the current optimizer attempt. Vector **e** (Fig. 5c) represents the residual translation of the distal bone segment. It runs from the centroid of the distal bone in the target position to the centroid of that same bone segment in the achieved position. The unit vector **k** defines the direction of the gravitation axis of the target bone and points in the distal direction (Fig. 5a,c). The term |**e** ⋅ **k**| therefore is the residual length error, *e*_*l*_, and |**e × k**| is the transverse translation of the distal bone segment (Fig. 5c). To quantify the transverse alignment of the proximal and middle bone assembly, we cannot simply use their centroid positions, since alignment can still be compromised if the proximal bone segment bends away from the target bone, or the middle bone segment is tilted, as shown by Fig. 5c. Parameter *e*_m_ is therefore assigned the average nearest neighbor distance between points of the middle and proximal bone segments, and points on the outer surface of the target bone. This value is 0 if the proximal and middle segments perfectly align with the target bone. Variable *b* (range [0, 1]) is also set by the user and balances between optimizing for transverse alignment of the distal segment (*b* = 0) or of the proximal and middle segments (*b* = 1).

### Software implementation

Custom software was written in the C +  + programming language (Visual Studio 2013, Microsoft, Redmond, WA), for preoperative planning^10,11^ and optimization of the parameters that control the oblique double-cut osteotomy procedure. The Visualization Toolkit^12^ (VTK 7.1.0) was used for 3-D visualization and Qt 4.8.6 was used for GUI programming^14^ (Nokia, Oslo, Norway).

#### Manual ODCRO’

After segmentation and position planning the user is enabled to position a proximal and distal plane to osteotomize the bone. The two plane orientations and the respective bone rotation angles are mutually linked by the rotation matrix **R**_c_ (Eq. 2). One plane and bone rotation should therefore follow the other. In the implementation of the software, the user is enabled to choose which plane and bone rotation angle should follow the other (Eqs. 2–4) (Fig. 6a, ‘Sync planes’ button). Subsequently executing the ODCRO causes the virtual bones to be cut at the specified locations followed by rotation of the respective bone segments. The result of this stitching procedure is visualized and represents the corrected bone.

#### ODCRO optimization

Alternatively, the user can choose to start the automatic optimization procedure to find a set of osteotomy parameters. This procedure is controlled by the metric defined by Eq. 5.

The user can again choose which plane orientation should follow the other, as described above, and then start the optimization procedure. At this point a dialog is shown (Fig. 6b) in which the user can influence the optimization procedure by choosing values for the balancing parameters *a* and *b* (Eq. 5a,b), represented by the sliders in Fig. 6b. Several dimensions in the 9-parameter search space can be deactivated (Fig. 6b, e.g., fix the in-plane translation of the distal (*x*_d_, *y*_d_) and/or proximal plane (*x*_p_, *y*_p_), fix the distal plane orientation (*φ*_d_, ψ_d_), translation (*d*_1_) or distal segment rotation (*β*_d_)), which limits the search space. The amount of overlap (*A*_min_), plane angulation (*α*_*max*_) and tissue torsion (*β*_max_) can finally be set to further tune the optimization procedure.

The optimizer stops iterating when a pragmatically set maximum number of iterations (1000), or metric tolerance (0.1 mm) is reached (Fig. 6b).

### Experimental evaluation of the method

All simulation experiments in this study are based on retrospective data containing 15 CT images of patients who were previously treated using conventional techniques in our academic hospital for a complex radius, tibia or femur deformity. All plans were implemented to evaluate our methodology but were not performed on these patients. These CT scans were acquired with standard patient protocols using a Brilliance 64-channel CT scanner (Philips Healthcare, Best, The Netherlands; isotropic voxel spacing 0.45 mm, 120 kV, 150 mAs, Pitch 0.6) and included the entire affected bone and healthy contralateral bone. All scans were anonymized. According to the Dutch Medical Research Involving Human Subjects Act, no approval of the medical ethics committee was required.

#### ODCRO vs OSCRO

One way to evaluate the alignment capabilities of our ODCRO method is to compare it with the achievements of a surgeon. However, since it is practically impossible to plan an ODCRO containing nine optimization parameters without computer assistance, the result would clearly be superior with the proposed technique. We therefore chose to compare the ODCRO method with the achievements of an OSCRO.

#### Lengthening

To investigate the ODCRO achievements in correcting for length, we select a subset of bones which required lengthening, and balanced, using parameter *a* (Table 1) the optimization parameters to improve length as good as possible. In this procedure we relaxed the amount of bone contact (*δ*_d_, *δ*_p_) (Table 2).

#### Error quantification

Alignment of an affected bone, the bone after OSCRO and after ODCRO is quantified by averaging the error distances (*d*_*err*_) of all the points in the corresponding polygon mesh to the nearest point in the polygon mesh of the target bone. This approach enables comparing the alignment of these three bone types quantitatively using a single parameter. When we aim to restore length as good as possible, we quantify the residual translation error (**e** in Eq. 5) in the length direction (*d*_*l*_ =|**e** ⋅ **k**|) and in the transverse direction (*d*_*t*_ =|**e** × **k**|).

#### Optimization time

An Asus Zenbook model UX331U (Asus, Taipei, Taiwan), with an Intel i7 – 8550U processor, 16 GB of RAM and an NVIDIA GeForce MX150 GPU (2 GB RAM) was used to evaluate the execution time of each optimization session. The optimization code was written in a single thread. The visualization toolkit ran on multiple threats.

## Results

**Figure 7:**
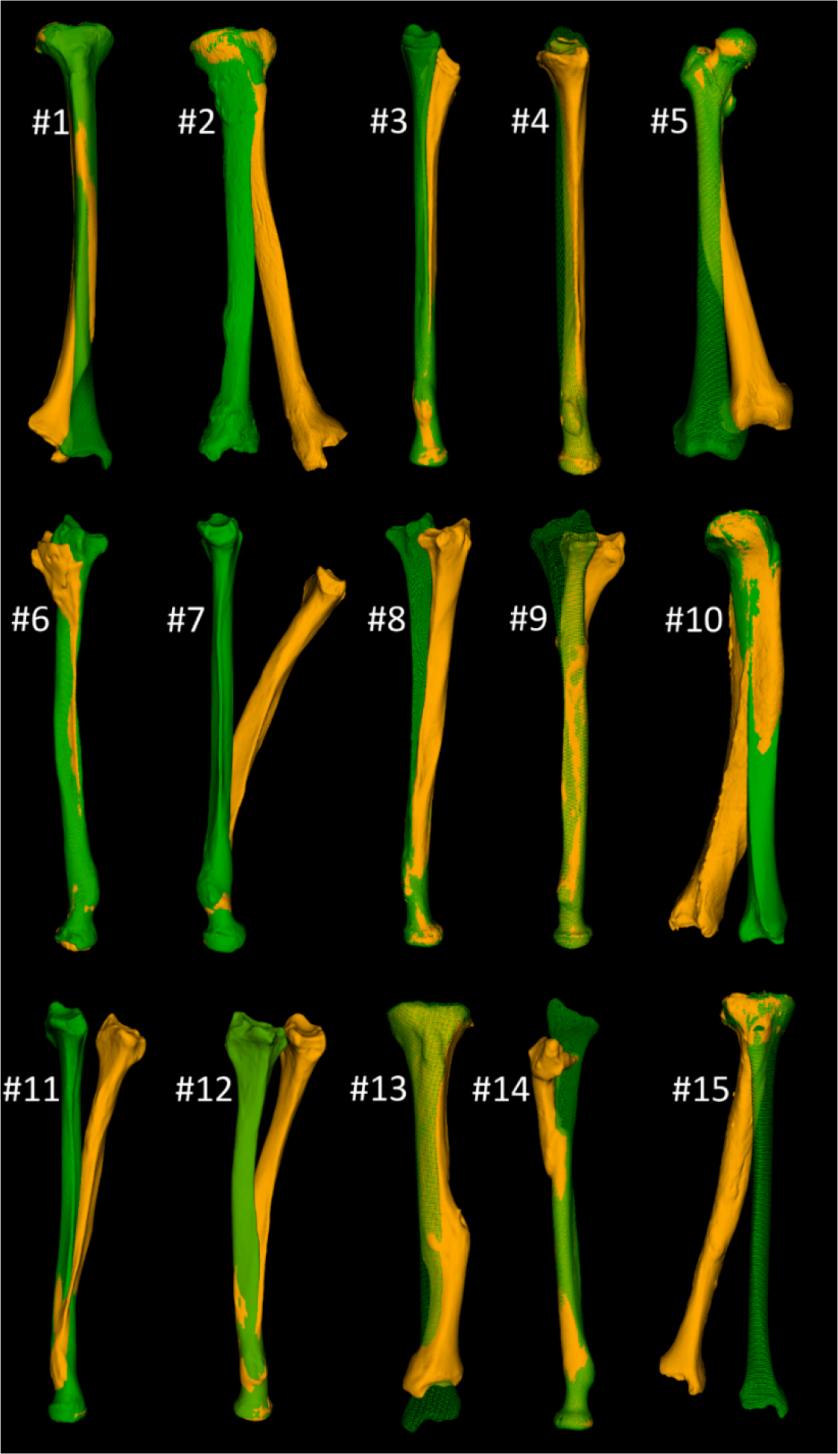
Deformed bone specimens (orange) of the radiuses, tibiae and femur included in this study. The mirrored contralateral bone (green) serves as reconstruction target.

**Figure 8:**
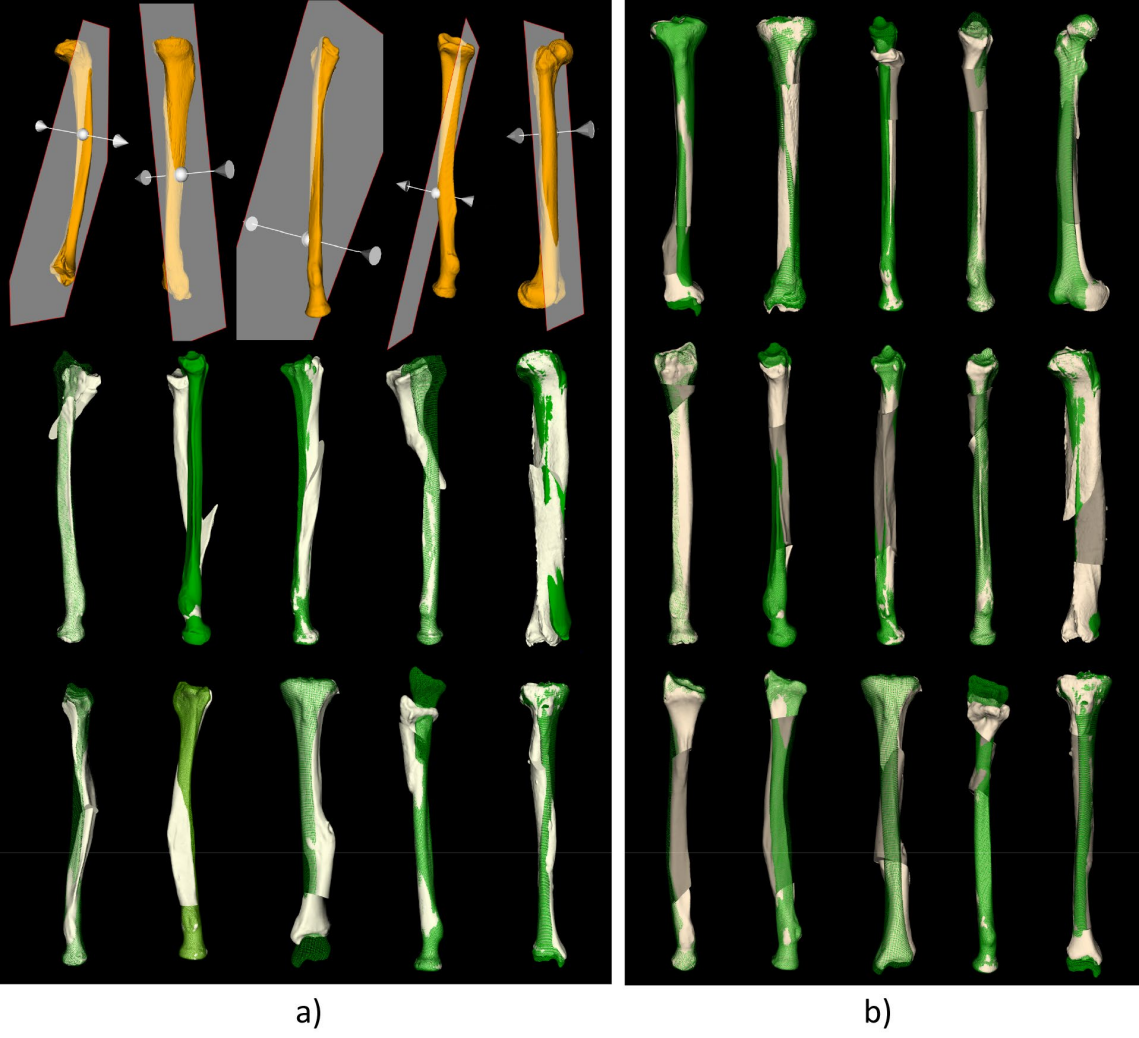
(**a**) Result of an oblique single cut rotation osteotomy (OSCRO) shown in white in comparison to the target bone (green). The top row shows cases where the osteotomy is too steep for clinical utilization. The remaining cases show perfect rotational alignment of the distal articular surface but a residual translation error and sometimes a large irregularity at the osteotomy location. (**b**) Resulting bone segments after an oblique double-cut rotation osteotomy (ODCRO) proximally aligned with the target bones (green) after running the optimization procedure balanced for length and transverse alignment (a = b = 0.5), 50% of bone overlap (*δ*_d_, *δ*_p_), maximum plane angulation (*α*_*max*_) 80 degrees.

Figure 7 shows the deformed bone specimens (orange) used in this study, proximally aligned with the target bone (green). Figure 8a shows attempts to reconstruct the deformed bones of Fig. 7 using an oblique single-cut rotation osteotomy^6^. The bones in the top row require a very steep osteotomy, which is not clinically feasible. The remaining cases perfectly restore rotational alignment of the distal articular surface at the cost of a residual translation error. In addition, the bones deviate from the target bone because of the deformation and sometimes show a large irregularity at the osteotomy location due to the required bone rotation in the OSCRO procedure.

The oblique double-cut rotation osteotomy was performed by first manually choosing the location of the osteotomy planes, the orientation of one plane and the corresponding bone rotation, while the orientation of the other plane and the accompanying bone rotation followed automatically. This manual procedure was repeated until the ODCRO result was close to the target bone, hereby narrowing the search space. The subsequent optimization procedure balanced length and transverse alignment (*a* = *b* = 0.5), respected 50% of bone overlap (*δ*_d_, *δ*_p_), and allowed a maximum plane angulation (*α*_*max*_) of 80 degrees (parameters shown in Fig. 6b). The results of optimization are visualized in Fig. 8b.

Compared to the single-cut approach of Fig. 8a, the oblique double-cut rotation osteotomy can restore those cases that are not eligible for treatment by an OSCRO (Fig. 8a, top row). The remaining cases benefit from the ODCRO approach as well, since the corrected bone (white) is generally better aligned with the target bone. The median time it took to complete the optimization procedure was 182 s (IQR 106–198 s) for radiuses and 408 s for tibiae and femur (IQR 338-701s).

**Figure 9:**
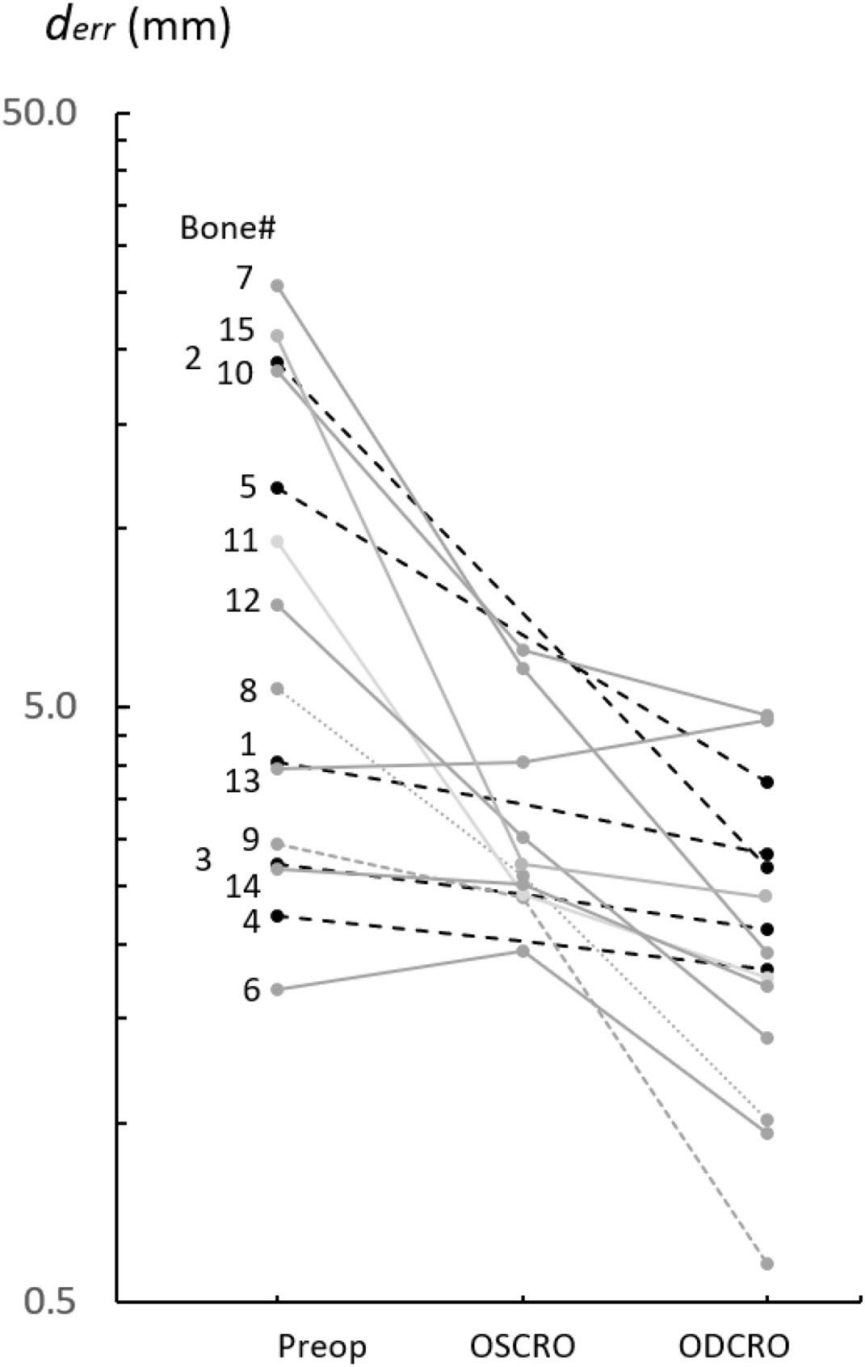
Malalignment before treatment (Preop), and after treatment using an oblique single-cut rotation osteotomy (OSCRO) or oblique double-cut rotation osteotomy (ODCRO), expressed in terms of the average distance (*d*_*err*_) of each point in the bone mesh (affected, corrected by OSCRO, corrected by ODCRO) to the nearest point in the target bone. Each line connects results for a bone case as shown in Figs. 7 and 8. The dotted lines represent cases that were not eligible for treatment by an OSCRO because of a very steep osteotomy.

The alignment of the affected bone, the bone after OSCRO and after ODCRO, as quantified by average nearest neighbor distance, *d*_*err*_, normally decreases after an OSCRO and decreases further after an ODCRO, as shown by Fig. 9, indicating better overall alignment. Only for bone #13 the overall alignment did not improve for an OSCRO or ODCRO. However, the bone length was restored to almost normal after an ODCRO (Fig. 8b).

**Figure 10:**
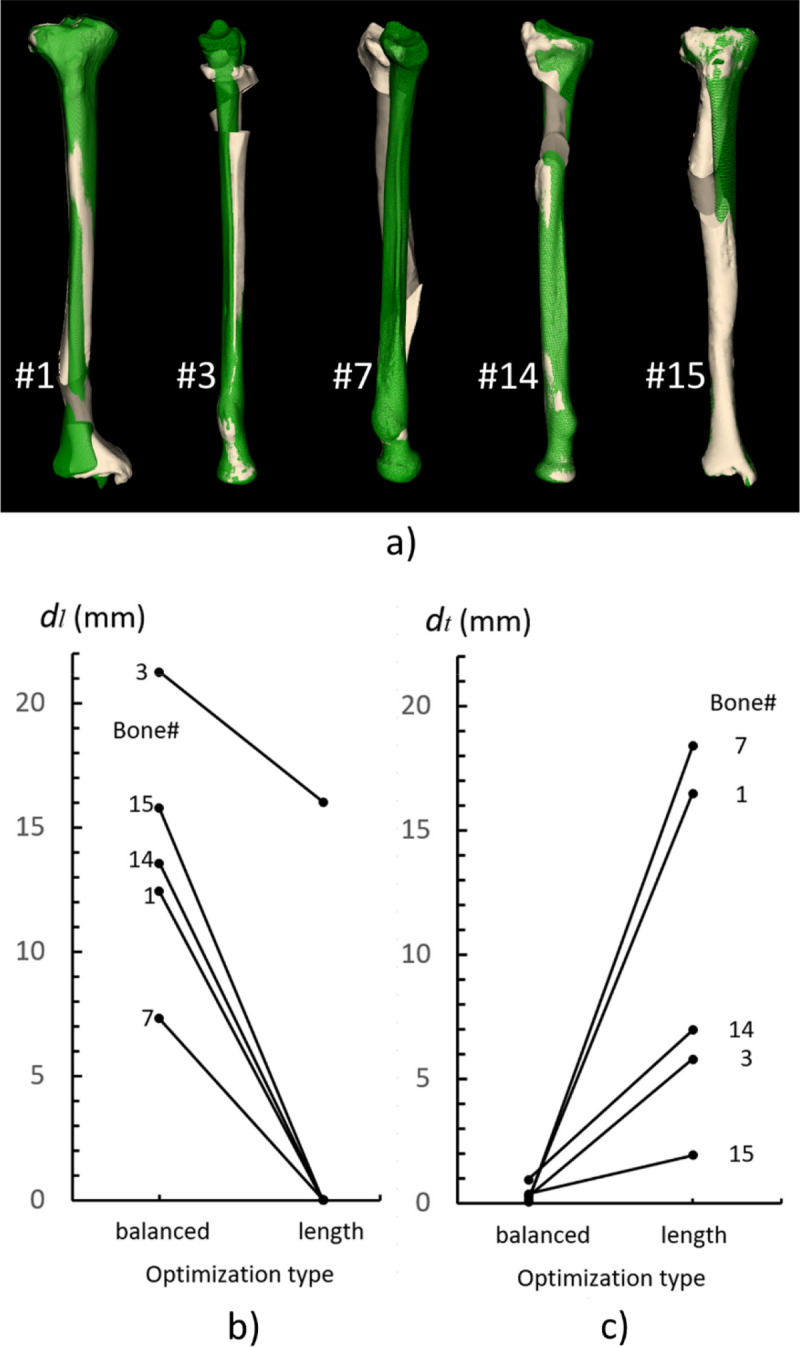
(**a**) Five bones selected from Fig. 8b, planned for treatment using an oblique double-cut rotation osteotomy, further optimized for length (*a* = [0.1–0.2], bone contact *δ*_d_ = *δ*_p_ = 0.2). The green bone represents the target. The residual length translation *d*_*l*_, and transverse displacement, *d*_*t*_, of the distal segment is quantified for (**b**) balanced ODCRO optimization as in Fig. 8b of the bone segments (*a* = 0.5) or (**c**) adjusted for optimal length (*a* = [0.1–0.2], bone contact *δ*_d_ = *δ*_p_ = 0.2) as shown in (**a**).

To further demonstrate the capabilities to correct for length, we selected bones (1, 3, 7, 14, 15) from Fig. 8b, because they required further lengthening, and balanced the optimization parameters to improve length as good as possible (*a* = [0.1–0.2]), at the cost of a deteriorated transverse alignment, while also relaxing the amount of bone contact (*δ*_d_ = *δ*_p_ = 0.2). Other optimization parameters were kept the same as above. Figure 10 visualizes the result, and quantifies the residual length error *d*_*l*_ (Fig. 10a) and transverse error *d*_*t*_ for the balanced approach (Fig. 10b) and after length optimization (Fig. 10c). It can clearly be seen that length is better restored at the cost of an increased transverse translation.

## Discussion

In this paper we introduced a method for preoperative planning of an oblique double-cut rotation osteotomy. The method is fairly straightforward considering the reduction of angular deformities although it requires an iterative optimization procedure to minimize residual translation deformity as well.

The oblique double-cut osteotomy has been reported to treat severely deformed and shortened lower limbs combined with distraction osteogenesis for three to six weeks^15^. It has also been reported to correct a curvature deformation of the humerus by means of two closing wedge osteotomies, leading to severe bone loss^16^. The use of a double-cut osteotomy has further been reported for treating torsional alignment syndrome of the knee by performing a straight osteotomy at the distal femur and proximal tibia and restoring anatomical alignment^17^. In these studies, in contrast to our method, no preoperative virtual 3D planning was used to keep the bone faces connected and to optimize for alignment. Instead, these procedures strongly rely on the surgeon’s skill in appreciating the deformity and restoring alignment^6^. In the technique that we propose, alignment planning is performed in six degrees of freedom, hereby minimizing angular and translational deformity in the coronal, sagittal and axial plane in one procedure, without additional bone loss due to wedge removal as in the case of a closed wedge osteotomy. The method enables controlling alignment of the bone segments with a reference bone by balancing optimization for length or transverse alignment of the bone segments.

This simulation study addressed the technique for preoperative planning. Transferring the plan to the actual patient’s bone, however, can be considered an additional challenge in which three bone segments have to be positioned in 3-D space. Different techniques have been described in the last decades, for navigating bone segments to the planned position. These techniques are either based on tool tracking^18,19^ or use patient specific instruments, such as cutting and/or drilling guides that fit the patient’s bone^20–24^, and reduction guides that bring the bone segments in the planned position^22^. Recent literature further reports on techniques that use patient-specific osteosynthesis material that force bone segments in the planned position while at the same time providing bone fixation^25^.

A limitation of optimization techniques is the fact that a suboptimal solution may be found at a local minimum^13^, especially in a multi-dimensional search space. We do not consider this a major limitation of our approach since solutions close to the global minimum may as well be acceptable for clinical reasons. The algorithm further enables us to manually choose osteotomy parameters and to quickly evaluate the result, before starting the automatic procedure. Fixing some parameters in the optimization procedure further helps narrowing the search space. In this study we did not perform a sensitivity analysis for the nine optimization parameters and for the balancing parameters *a* and *b*, which may be considered a limitation. We recommend performing a sensitivity analysis in future implementations of our approach. A disadvantage of the method is that it is relatively slow to find an optimal solution. Partly because of the manual initialization, and partly due to the time it takes for the algorithm to complete the optimization. However, for practical reasons we evaluated the method and its execution speed of the ODCRO procedure on a mobile ultrabook. There are several ways to speed up the procedure, such as using a high-end workstation or reduction of the polygon meshes representing the bones. In our approach the user controls optimizing for length and transverse displacement using balancing parameters *a* and *b*. For future implementations it may be of interest to investigate using a multi-objective approach^24^ in which the user may select two objectives, e.g., optimizing for length *and* transverse displacement. After optimization the user could make a selection out of a set of pareto-optimal solutions.

In this study we used typical examples of seriously deformed bones to demonstrate the methodology from a technical, and not from a clinical point of view. In actual patient cases a surgeon may choose to only allow cutting a bone in a specific region for clinical reasons. Or to limit the osteotomy plane angle or bone rotation. These constraints are supported by the proposed method although they may compromise the resulting plan. The method should be considered a tool that helps in planning the complex ODCRO approach, while the surgeon still needs to judge whether the proposed result is clinically feasible. In one of our previous studies the ODCRO procedure already showed to be clinically feasible for clavicular reconstruction^26^.

## Conclusion

Choosing adequate osteotomy parameters in the application of an ODCRO is extremely difficult without computer assistance. The proposed method restores rotational alignment and optimizes translational alignment using an iterative procedure. The proposed ODCRO procedure has shown to be effective for different bone types where the single-cut approach fails. It further yields a better alignment of bone segments with a target bone, which renders the method of interest for future corrective surgery.

## Appendix, rotation about an arbitrary line

The rotation matrix that rotates an object over an angle *β*_d_ about an arbitrary line (or helical axis) through the origin in 3-D space, is found^27^ by rotating space about the z-axis to bring the line into the xz-plane. Next, space is rotated about the y-axis until the line is along the z-axis. In this state the object can simply be rotated about the line, i.e. the z-axis, using a straightforward rotation matrix **R**_z_(*β*). To bring the rotated object to the original line position in 3-D space, it is rotated back about the y-axis and z-axis. These consecutive steps are combined in a single rotation matrix **R**_d_(**p**_d_, **n**_d_, *β*_d_):

6 $${\mathbf{R}}_{{\text{d}}} ({\mathbf{p}}_{{\text{d}}} ,{\mathbf{n}}_{{\text{d}}} ,\beta_{{\text{d}}} ) = {\mathbf{R}}_{{\text{z}}} (\varphi_{{\text{z}}} ){\mathbf{R}}_{{\text{y}}} (\varphi_{{\text{y}}} ){\mathbf{R}}_{{\text{z}}} (\beta_{{\text{d}}} ){\mathbf{R}}_{{\text{y}}} ( - \varphi_{{\text{y}}} ){\mathbf{R}}_{{\text{z}}} ( - \varphi_{{\text{z}}} )$$

with:

**p**_d_ = (*p*_x_, *p*_y_, *p*_z_), a point on line *l*. **n**_d_ = (*n*_x_, *n*_y_, *n*_z_), unit direction vector of line *l*. *β*_d_ = angle of rotation about line *l*

7 $${\varphi }_{z}=atan\frac{{n}_{y}}{{n}_{x}}{; \varphi }_{y}=atan\sqrt{\left({n}_{x}^{2}+{n}_{y}^{2}\right)}/{n}_{z}$$

8 $${\mathbf{R}}_{z}\left({\alpha}\right)=\left[\begin{array}{ccc}Cos& -Sin& 0\\ Sin& Cos& 0\\ 0& 0& 1\end{array}\right]; {\mathbf{R}}_{y}\left({\varphi }_{y}\right)=\left[\begin{array}{ccc}Cos{\varphi }_{y}& 0& {Sin\varphi }_{y}\\ 0& 1& 0\\ -Sin{\varphi }_{y}& 0& Cos{\varphi }_{y}\end{array}\right]$$

With *α* being *φ*_z_ or *β*. In case the arbitrary line *l*:**x** = **p**_d_ + λ **n**_d_ does not go through the origin, which is normally the case in a rotation osteotomy, space can first be translated using translation matrix **T**(-**p**_d_) before applying rotation matrix **R**_d_. Finally space is translated back to the original location using **T(p**_d_).

## References

[1] Patton MW (2004). Distal radius malunion. J. Am. Soc. Surg. Hand..

[2] Disseldorp DJG, Poeze M, Hannemann PFW, Brink PRG (2015). Is bone grafting necessary in the treatment of malunited distal radius fractures?. J. Wrist Surg..

[3] Wieland AWJ, Dekkers GHG, Brink PRG (2004). Open wedge osteotomy for malunited extraarticular distal radius fractures with plate osteosynthesis without bone grafting. Eur. J. Trauma.

[4] Dobbe JGG, Du Pré KJ, Kloen P, Blankevoort L, Streekstra GJ (2011). Computer-assisted and patient-specific 3-D planning and evaluation of a single-cut rotational osteotomy for complex long-bone deformities. Med. Biol. Eng. Comput..

[5] Sangeorzan BP, Judd RP, Sangeorzan BJ (1989). Mathematical analysis of single-cut osteotomy for complex long bone deformity. J. Biomech..

[6] Dobbe JGG, Strackee SD, Streekstra GJ (2018). Minimizing the translation error in the application of an oblique single-cut rotation osteotomy: Where to cut?. IEEE Trans. Biomed. Eng..

[7] Peymani A (2018). Surgical management of madelung deformity: A systematic review. Hand.

[8] Schkommodau E (2005). Computer-assisted optimization of correction osteotomies on lower extremities. Comput. Aided Surg..

[9] Vroemen JC, Dobbe JGG, Strackee SD, Streekstra GJ (2013). Positioning evaluation of corrective osteotomy for the malunited radius: 3-D CT versus 2-D radiographs. Orthopedics.

[10] Dobbe JGG (2011). Computer-assisted planning and navigation for corrective distal radius osteotomy, based on pre- and intraoperative imaging. IEEE Trans. Biomed. Eng..

[11] Dobbe JGG, De Roo MGA, Visschers JC, Strackee SD, Streekstra GJ (2019). Evaluation of a quantitative method for carpal motion analysis using clinical 3-D and 4D CT protocols. IEEE Trans. Med. Imag..

[12] Schroeder W, Martin K, Lorensen B (2006). The Visualization Toolkit, An Object-Oriented Approach to 3D Graphics.

[13] Press WH, Teukolsky SA, Vettering WT, Flannery BP (2007). Numerical Recipes: The Art of Scientific Computing.

[14] Blanchette J (2006). Summerfield M, C++ GUI Programming With Qt 4.

[15] Yadav SS (1993). Double oblique diaphyseal osteotomy. J. Bone Jt. Surg. Br..

[16] Brown GA, Firoozbakhsh K, DeCoster TA, Reyna JR, Moneim M (2004). Rapid prototyping: Future of trauma surgery?. J. Bone Jt. Surg..

[17] Leonardi F, Rivera F, Zorzan A, Ali SM (2013). Bilateral double osteotomy in severe torsional malalignment syndrome: 16 years follow-up. J. Orthopaed. Tramatol..

[18] Athwal GS, Ellis RE, Small CF, Pichora DR (2003). Computer-assisted distal radius osteotomy. J. Hand Surg..

[19] States RA, Pappas E (2006). Precision and repeatability of the Optotrak 3020 motion measurement system. J. Med. Eng. Tech..

[20] Ciocca L, De Crescenzio F, Fantini M, Scotti R (2009). CAD/CAM and rapid prototyped scaffold construction for bone regenerative medicine and surgical transfer of virtual planning: A pilot study. Comput. Med. Image Graph.

[21] Dobbe JGG, Vroemen JC, Strackee SD, Streekstra GJ (2013). Corrective distal radius osteotomy: Including bilateral differences in 3D planning. Med. Biol. Eng. Comput..

[22] Murase T (2008). Three-dimensional corrective osteotomy of malunited fractures of the upper extremity with use of a computer simulation system. J. Bone Jt. Surg. Am..

[23] Miyake J, Murase T, Moritomo H, Sugamoto K, Yoshikawa H (2011). Distal radius osteotomy with volar locking plates based on computer simulation. Clin. Orthop. Relat. Res..

[24] Carrillo F (2020). An automatic genetic algorithm framework for the optimization of three-dimensional surgical plans of forearm corrective osteotomies. Med. Image Anal..

[25] Dobbe JGG, Vroemen JC, Strackee SD, Streekstra GJ (2014). Patient-specific distal radius locking plate for fixation and accurate 3D positioning in corrective osteotomy. Strateg. Trauma Limb Reconstr..

[26] Grewal S, Dobbe JGG, Kloen P (2018). Corrective osteotomy in symptomatic clavicular malunion using computer-assisted 3D planning and patient-specific surgical guides. J. Orthoped..

[27] Murray, G., Rotation matrices and formulas, https://sites.google.com/site/glennmurray/Home (2013)

